# Motherhood challenges and well-being along with the studentship role among Iranian women: A qualitative study

**DOI:** 10.1080/17482631.2017.1335168

**Published:** 2017-06-19

**Authors:** Zahra Behboodi Moghadam, Maryam Ordibeheshti Khiaban, Maryam Esmaeili, Mahvash Salsali

**Affiliations:** ^a^ Department of Reproductive Health, School of Nursing and Midwifery, Tehran University of Medical Sciences, Tehran, Iran; ^b^ Department of Critical Care Nursing, School of Nursing and Midwifery, Tehran University of Medical Sciences, Tehran, Iran; ^c^ Department of Medical and Surgical Nursing, School of Nursing and Midwifery, Tehran University of Medical Sciences, Tehran, Iran

**Keywords:** Content analysis, experiences, female students, student mothers, Iran, motherhood, qualitative research

## Abstract

This study purposed to explore and describe the experiences of Iranian female students with the role of motherhood. This 2015 qualitative study used purposeful sampling to select 20 student mothers aged 24–50 who were studying at a state or non-state university in an urban area in northwest Iran. Data was collected through individual semi-structured interviews and analysed using a qualitative content analysis approach. Three main themes were developed during data analysis: “simultaneous management”, “facilities”, and “barriers”. The management of maternal and family affairs by female students in universities where motherhood is not supported is a challenge. The significance of mother-student roles must be emphasized and support and education provided for women to gain skills useful in playing these roles. Policy makers should devise strategies for bringing change to the traditional perspective that motherhood and educational responsibilities cannot be met at the same time by one person.

## Introduction

The number of student mothers entering universities has been increasing around the world since 1966 (Williams, Alon, & Bornstein, 2006) and since 2001 in Iran where females compose the majority of university students (Zahedifar, 2012). The age of the majority of female students corresponds with their reproductive age (Springer, Parker, & Leviten-Reid, 2009). Therefore, the existence of student mothers raises concerns about playing the roles of mother and student (White, 2008). A woman may enthusiastically embrace the simultaneous roles of mother and student; however, undertaking these two roles, even in ideal conditions, can pull one person in two directions (Springer et al., 2009).

Combining motherhood and studying without compromising the activities of either one is a great dilemma for student mothers. When a woman must focus all her attention on her studies, her behaviour may contrast with her traditional motherhood role (Visick, 2009). While discourse regarding the “good mother” in any society is based on the traditional motherhood role (Goodwin & Huppatz, 2010), its definitions vary by society given the different experiences and challenges of motherhood in diverse cultures (Zhang, 2011). Role challenges cause women to abandon one role for the sake of the other (Springer et al., 2009). Myths, expectations, and ideals available in the campus culture can influence this behaviour.

Academic activities are intertwined with challenging competitions. Therefore, motherhood responsibilities impose a large burden on students’ shoulders. The academic community focuses mainly on success, development, and never-ending competitions without providing any support. Therefore, taking on motherhood along with studies is not considered normal in universities. Student mothers experience unpleasant emotional pressures and receive negative feedback from the academic setting, implying that education is the first priority. Moreover, prejudice towards student mothers and the labelling of them as non-productive stimulate avoidance behaviours and a discriminatory allocation of educational resources to other students (Springer et al., 2009). Therefore, student mothers avoid bringing their child with them or hide their parenting roles (Williams et al., 2006). From an academic’s perspective, bringing a child indicates that the student mother does not have the required interest and enthusiasm to take the required steps for scientific development (Adofo, 2013).

Education is undoubtedly a source of empowerment and development. Achieving development goals in any society depends on women’s participation in education (Esia-Donkoh, 2014). Education is a starting point for life improvement and has a significant impact on family members (Adofo, 2013). It should be noted that the education of women is required to achieve the Millennium Development Goals (MDGs) of gender equality and empowerment. Achieving the MDGs’ goal is an unfinished agenda and requires comprehensive and transformative methods with a new development framework (UN Women, 2013).

Education is recognized as a developmental tool and a female individual’s right. Nevertheless, the characteristics of female reproduction have become a barrier to some female students’ achievement of their full potential in the academic setting. A woman’s reproduction rights, such as having the right to have children, are not considered equally as important as their education rights. It is essential that females’ reproduction rights are recognized in academic settings and their rights for education are respected with consideration given their demographic characteristics (Esia-Donkoh, 2014). Iranian student mothers with different contexts and cultures have special needs. The current study purposed to explore and describe the experiences of Iranian student mothers. The findings can be used to develop educational policies to facilitate the provision of appropriate healthcare services to this group of students. In addition, the findings can introduce a discourse for female individuals who seek a balance between studying and motherhood.

## Methods

### Design

This qualitative study used a content analysis approach. Qualitative studies aim to improve the understanding of phenomena through human experiences (Myers, 2000). According to Grbich (2007), content analysis is a systematic process of coding large amounts of textual data for determining trends and themes (Grbich, 2007).

### Data collection and participants

Data was collected from April to December, 2015 through in-depth semi-structured interviews held with 20 student mothers who were studying at the state and non-state universities in Tabriz (an urban area in northwest Iran).

Participants were chosen through purposeful sampling based on their lived experiences with the study phenomenon; consideration was given the maximum variations in sampling in terms of demographic characteristics (Table I). Being a student and a mother at the same time was the selection criteria. Nine of these participants were employed and continuing their education due to recent evolutions in the global economy, to attain more skills, meet employers’ expectations, improve their revenue-making capacities, further contribute to their families’ life expenses, and to create a better life for themselves and their children (Adofo, 2013; Forster & Offei-Ansah, 2012; Leaman, 2015). In addition, mothers tend to be employed to supply their education expenses (Erk, 2013), or see employment as a choice which will enhance their social position (similar to education) (Dunifon & Gill, 2013; Moinifar, 2011). To accommodate maximum diversity, no limitation was set on the children’s ages as an entrance criterion. Most student mothers in graduate programmes were middle-aged (Springer et al., 2009) and their children were often in the stages of adolescence or youth. Challenges associated with mothering, however, are always present despite differences in the children’s ages (Acton, 2009; Lehr, 2005; Shahhosseini, Simbar, Ramezankhani, & Alavi Majd, 2012).

**Table I.** The socio-demographic characteristics of the participants.

Characteristics	*N*
*Educational discipline*	
Medical sciences	12
Non-medical sciences	8
Basic sciences	**3**
Engineering	**2**
Human sciences	**3**
*Educational level*	
Bachelor degree	8
Master degree	8
Professional doctoral degree	2
Ph.D. degree	2
*University*	
State	10
Private	10
*Age (years)*	
20–30	8
31–40	8
41–50	4
*The number of children*	
1	14
2	6
*Children’s age (years)*	
Less than 1	1
1–6	7
7–11	8
12–18	2
More than 18	2
*Residence*	
Native	6
Non-native	14
*Occupation*	
Employed	9
Unemployed	11
*Husband’s educational level*	
Bachelor degree	6
Master degree	10
Professional doctoral degree	4
*Financial condition*	
Intermediate	7
Good	13

After obtaining the required permission from the authorities, the names, academic disciplines, and contact information of all students in the research zone were obtained. Their educational files were reviewed to determine those students who were also mothers. Then, the student mothers were contacted, informed of the study’s aim, and asked to provide some details of their family life. Appointments were scheduled with those students who agreed to participate in this study at a time and place convenient to them. An interview guide was adopted by the research team before the interviews. The corresponding author (M.O.) performed pilot interviews and asked other team members to give feedback regarding their quality. The interview guide consisted of open-ended questions regarding the study phenomenon. The major foci of the questions asked during interviews were as follow:

- What is your experience of being a mother and student at the same time?
- How do you play your roles as a mother and student at the same time?
- How do you feel about being a mother and student at the same time?

In order to enhance the reliability and quality of the first interview, another interview was conducted with the same participant one week later. Most interviews were conducted in a private room at the university where the participants studied (18 interview sessions) and in the presence of the interviewer and the participant only. Two interviews were held in the participants’ workplaces, and one was conducted in the participant’s home. The interviews were continued until data saturation was reached and no new concept was developed from the collected data. Interviews were held in Farsi and then translated into English by a bilingual translator for publication purposes. A bilingual translator ensured the quality of the translation for preserving meanings and concepts. The interviews were all recorded with a recorder except one; the participant agreed to tape-record her voice using her own recorder to ensure the accuracy and adequacy of notes taken during this interview session. The duration of the interviews was 40–90 min with an average of 65 min.

### Data analysis

Immediately after each session, the interviews were transcribed verbatim and read several times to get the sense of the whole, and the data was analysed using the method suggested by Graneheim and Landman (2004). Meaning units as phrases and sentences related to the experience of motherhood were determined. The related meaning units were labelled with codes and sorted into categories and subcategories based on their similarities and differences. Lastly, similar categories were abstracted and labelled with themes and subtheme indicating there was a latent meaning in the text. The MAXQDA software version 10 was used to help with data classification and management during analysis.

### Rigor

The criteria suggested by Lincoln and Guba ( 1985) were used to ensure the rigour of this study. They included credibility, transferability, dependability, and confirmability (Denzin & Lincoln, 2005). Credibility was strengthened through the prolonged engagement with the samples of two researchers (MO and ZB) who remained continuously throughout data collection. Also, discussions among research team members regarding the findings helped with this part of rigour. Other considerations were member checking, peer checking, and long engagement with the collected data. For member checking, the participants were asked to read a brief report containing transcripts and initial findings and send feedback to ensure that their real thoughts were reflected. Three qualitative researchers in the field of reproductive health were asked to independently analyse the data using the suggested method applied in this study, and their data analysis results were compared with the authors’ findings, which led to the modification of some data. In case of a disagreement, discussions were held to reach a consensus. The researchers remained engaged with the participants for an extended period so as to gain their trust and collect in-depth data. Transferability was enhanced through purposive and maximum variation in sampling in terms of demographic characteristics. A detailed description of the data collection and analysis processes was provided. Dependability was strengthened by engaging more than one researcher in the data analysis process. In other words, one author collected and analysed the data (M.O.), and two other researchers (Z.B. and M.E.) discussed, checked, and verified the findings. Confirmability was enhanced by keeping an easy to follow audit trial of all research activities, methodological decisions, and analysis notes.

### Ethical considerations

This study was one part of the corresponding author’s (M.O.) Ph.D. thesis in the field of reproductive health. The approval of the ethics committee affiliated with the author’s institution was received in April, 2015 (decree number = 9121151003). Some of the ethical principles that should be considered in qualitative research include obtaining informed consent from the participants and respecting the anonymity and confidentiality of the findings (Sanjari, Bahramnezhad, Khoshnava Fomani, Shoghi, & Cheraghi, 2014). In this research, all participants were informed of the purpose and methods of the study, and permission for tape-recording the interviews and publishing the findings was obtained from the participants. The participants were informed of their rights and the voluntary nature of participating in this study, their anonymity, and the confidentiality of the data. Participants were ensured that withdrawal from the study was possible without being penalty. Those who wished to participate in this study signed a written informed consent form.

## Findings

Three main themes were developed during the data analysis: “simultaneous management”, “facilities”, and “barriers” (Table II). Each theme is described below, and participant quotations are given.

**Table II.** Themes and sub-themes developed during the data analysis.

Themes	Sub-themes
*Simultaneous management*	Planning Sacrificing
*Facilities*	Self-efficacy Comprehensive support
*Barriers*	An inappropriate social and financial condition The inflexible education system Physical and mental strain

### Simultaneous management

This theme consisted of two sub-themes: “planning” and “sacrificing”. Almost all student mothers had to increase their control over situations in order to fulfil their multiple tasks as mother, student, housewife, and so on. They applied various techniques such as “planning”. However, when duties overlapped, motherhood tasks took priority. In other words, they sacrificed to conduct familial duties.

#### Planning

All participants made plans to coordinate and organize their motherhood and student duties. For instance, they made efforts to organize home affairs, motherhood tasks, work-related duties, and financial affairs.

Participant A said:

In a movie, the actor said that the mother’s mind is like the airport control tower. If there is any problem in planning for airplanes, a dreadful accident may happen. I believe in such a perspective. I provide the list of my tasks that should be done during a day, week, and month and prioritize them so I do not forget my tasks and duties and accidents are prevented!

The most important principle in the planning of motherhood duties emphasized by the participants was “seeking an appropriate child-minder”. In this regard, Student B stated:

When my kids were little, I could not send them to kindergarten or get a child-minder. So, I had to take a leave of absence from the university for seven months and take care of my child. When my child was seven months old, I had to get a baby-sitter so I could go to the university. When I had examinations, the baby-sitter was working part-time and I had to leave my child with my mother-in-law, who lived in a nearby city, for twenty days. It was very hard for both of us.

Financial affairs also needed some sort of planning and organizing so that educational costs could be paid. Participant C said:

Since I became a student, my family has had to decrease the budget for leisure and shopping, because they must pay my tuition fee.

#### Sacrificing

Participants planned, had multiple responsibilities, and faced unpredictable situations. Therefore, they had to prioritize their duties and mainly “preferred their family and children over their studies”. For example, Student D said:

My son was sick and had a fever. I had to take a final examination. My husband had gone on a business trip. I could not leave my child alone, nor could I leave the examination; I was at a crossroad. Finally, I left the examination, because my child was more important to me.

Another point made by participants was “use of spare time”. Student mothers spent their time at home on children and family affairs. Therefore, they had to study when other family members were unavailable or asleep, which reduced the quality of their educational tasks. Participant E said:

When all family members are asleep, I turn on the computer and start my night life. I make less noise and do not disturb others.

### Facilities

This theme consisted of two sub-themes: “self-efficacy” and “comprehensive support”. Facilities refer to the quality which simplifies and optimizes the performance of different tasks. In the present research, facilities means all factors contributing to the simple and optimized simultaneous performance of mothering and studying at university, which were perceived by the participants and most frequently referred to in the interviews. In terms of facilities, the findings of the present research can be categorized into three scopes: dispositional, situational, and institutional.

#### Self-efficacy

In talking about the coordination and management of their mothering role along with their role as a student, the participants referred to such characteristics as responsibility, determination, and confidence. These characteristics are among the dimensions of self-efficacy which is one of dispositional facilitators. For instance, Participant F said:

When I got pregnant with my second child, one of my professors at the university told me that, even though I had been a good student, parenting is not an easy task to do and I was unlikely to be able to continue my studies at higher levels. I was pretty confident that I could manage to be both an outstanding mother and a successful student; and that is what I actually achieved today.

Moreover, some participants recognized marriage, mothering, and associated experiences as contributing to enhanced self-efficacy. Student G reported:

Not only do I manage in-house and parenting tasks way better than many housewives, my educational state is also way better than that of single students. I am also my class representative and have activities in the Scientific Association of the university and its journal. I feel that my marriage and mothering have made me even faster and have added to my tolerance, making me stronger and more responsible than the single students.

#### Comprehensive support

All participants strongly believed that receiving support for emotional, financial, mothering, housekeeping, and educational aspects of life facilitated taking roles and responsibilities. Participant H said:

My sister played the main role in my educational success. She encouraged me to study. She helped me with childcare, lent me money, and always reminded me of her support at all times and under all conditions.

Support may come from the student’s husband, family members, university professors, or classmates. Based on the support source, these factors are categorized into situational or institutional facilities. Student I declared:

My spouse was very helpful. He used to say, ‘Don’t worry about lunch and dinner; I’ll have biscuits.’ He was a great help. I did not give him biscuits to eat, but he had no problem with just a simple meal and was not strict.

### Barriers

In the present research, “barriers” refers to all factors and conditions which tend to limit or raise problems for simultaneously playing the roles of mother and student; in the context of the present research, these have been perceived by the participants and exhibited the highest frequency in the findings. This theme consisted of three sub-themes: “an inappropriate social and financial condition”, “the inflexible education system”, and “physical and mental strain”. The barriers found in the present research fell within either of three scopes: dispositional, situational, and institutional.

#### An inappropriate social and financial condition

This sub-theme falls within the scope of situational barriers. Most participants described inappropriate social and financial conditions as “financial difficulties” and “a lack of family support” for various reasons and “having a dependent child”. “A lack of family support” due to family disputes was also emphasized by the participants. The strict and controlling behaviour of husbands was a cause for family issues. Student J said:

One day I was in the classroom when my husband called and asked to use my car. I said, ‘You can come and take it.’ He came to the university and stood outside the classroom door. He looked inside the classroom and found that ten male students and I were sitting there listening to the instructor. He got upset and said, ‘I did not know about this situation. Do you think it is really worth it? You have left everything and are sitting here among ten men. You cannot study like this.’

The stereotypical image of the mother was mentioned by the participants with a focus on “a lack of family support”. Based on “the social stereotypical image of the motherhood role”, a good mother was seen as a housekeeper who was always available for her children and spent all her time with them. Those mothers who behaved otherwise seemed to be bad and selfish and were accused by society. Student K said:

Sometimes when my kids had problems and I could not respond to them due to my studies and time limitations, I seemed selfish. For instance, once my daughter’s teacher called me regarding her school work and invited me to the school. I said that I couldn’t come, because I was busy. Then she said, ‘Well, your child is more important is she not?’ It was really embarrassing.

“The inappropriate socio-economic conditions” and economic issues were mentioned by all participants. Even though many of the student mothers in this study enjoyed a relatively good financial situation, the costs associated with transportation, dormitory rental, ready-to-serve food, and child care were seen to either set limits for the family expenditures on recreational activities or force the family to spend their savings or sell their assets, such as their house. Participant L said:

We had a good financial situation, and soon my husband and I could buy a house. Both of us were accepted at university and had to pay for tuition fees. We decided to sell the house to pay for expenses, but we knew that we might not be able to do so in the future.

For this reason, some student mothers had no choice but to continue working while educating or to seek a part-time job to meet the primary and secondary costs associated with their education; this raised more challenges for the mother herself as well as the whole family. In this respect, Participant M said:

Even following my admission to the university, I couldn’t leave my job, because the family’s life expenses were increased by my education. I hate having to always look explanations for my boss, professor, child, husband, mother, or friends as to why I can no longer accomplish what they expect me to do.

#### The inflexible education system

The participants referred to the subtheme of “the inflexible education system” as an institutional barrier to taking on the roles of both mother and student. “Inflexible rules”, “inappropriate curricula”, “a lack of facilities for non-native students”, and “limited collaboration of instructors and classmates” were the aspects of the results. Student N declared:

Since I was employed and my husband was away, I asked my teachers to excuse my late arrivals or absences. Some of them said, ‘It is your problem and you must sort it out yourself. You had to solve problems; otherwise, you would not have permission to continue your studies.’ When I was late for a class, my teachers would tell me they were going to reduce my final grade because of being late. I accepted it as I had no other choice.

#### Physical and mental strain

All participants considered the subtheme of “physical and mental strains” as the most important dispositional barriers to the simultaneous management of the roles of mother and student. “Desperation”, “great stress”, “nostalgia”, “guilt”, and “fatigue and physical pressure” were highlighted by participants. The student mothers suffered from fatigue caused by work pressures and multiple roles. Student O stated:

I tolerate so much pressure. When I go home at night after classes, I have to do motherly tasks and prepare food. When the work is done and I want to study, I am too tired. Wherever I sit, right there, I go to sleep, and my husband wakes me up to go to bed.

Participants also experienced so much stress and constant worry about their children and their studies. They felt selfish for not looking after their children properly. Student P said:

I had obsessive thoughts for a long time, when I was drowned deep in my studies. I was suddenly wondering what if something happened to my child? I would have to endure a life full of regret. I had a neighbour who was a teacher and she went to the university for higher education. That poor teacher! One day when she was at the university, her son was cycling when a car hit him and he died. Do you think that the mother would ever forgive herself? After that tragedy, is life with everything in it worth it? I became obsessed and I could not concentrate. Right in the middle of work, I was wondering what if something happened to my child? What could I do then? I started praying, ‘God I have given my child to you; take care of him and please do not disappoint me.’

Moreover, regarding a sense of guilt for neglecting his children, Student Q expressed:

This year, my child was getting ready for the nationwide university entrance exam (Konkoor), during which time most parents accompany their children to emotionally support them. However, I was busy travelling between my city of residence and the city in which I was educating, leaving my child home alone; accordingly, I will never forgive myself should he fail to be admitted to his major of interest.

## Discussion

This study aimed to explore and describe the experiences of Iranian student mothers with the role of motherhood. The results indicate that simultaneously being a student and a mother was challenging, yet manageable, requiring planning in various fields. Moreau and Kerner (2013) stated that the nature of parenting and academic tasks required careful planning to combine these activities (Moreau & Kerner, 2013). According to Adofo (2013) to appropriately perform multiple roles, student mothers in Ghana applied simultaneous management strategies and organization approaches to adapt to contradictions resulting from concurrent tasks (Adofo, 2013). Similarly, Forster and Offei-Ansah (2012) conducted a study entitled Domestic affairs and coping strategies of female students in Ghana. In their study, students used a variety of strategies, such as delegating domestic roles, prioritizing, planning, and organizing activities to ensure that their family life did not suffer while they were at university (Forster & Offei-Ansah, 2012).

In this study, one of the most important aspects of planning for motherhood roles was the selection of an alternative method for childcare. Berg and Mamhute (2013) quoted from Mendes and stated that without proper childcare, taking on the student role becomes very difficult for young mothers (Berg & Mamhute, 2013). In a study entitled The challenges and adaptive mechanisms of nurse student mothers, Adofo (2013) suggested that the use of measures such as recruiting and hiring workers to do housework and childcare or leaving children at a kindergarten or with grandparents were helpful (Adofo, 2013).

The results of this study showed that whenever the roles of mother and student overlapped, student mothers made their families and children a priority over their educational duties. The results of this study were in line with those of Forster and Offei-Ansah (2012), who determined that giving priority to the family and responsibilities of marital life created problems for doing academic tasks, because the comfort of the family and children was more important than studies for female students (Forster & Offei-Ansah, 2012). In a study entitled College students as mothers, Erk stated that U.S. student mothers made many sacrifices to overcome obstacles and achieve success (Erk, 2013). One example of sacrifice mentioned by the participants was the need to spend time with a sick child at the cost of losing educational goals. Other studies have confirmed that one cause of emotional turmoil and stress for student mothers was their child’s illness. Almost all student mothers stated that the fear of losing a child to illness was so great that they stayed with their ill children all the time until they got well, even if it prevented them from attending to academic tasks (Esia-Donkoh, 2014). Adofo (2013) also stated that student mothers had to look after their sick children; therefore, they could not prepare for examinations and often did not pass them successfully (Adofo, 2013).

Fuller and Paton (2007) found that social and personal conditions can act as either facilitators or barriers against the decision to continue to educate (Fuller & Paton, 2007; Smith, 2008). Mercer (1993), Fairchild (2003), Hayes Nelson (2009), and Flynn, Brown, Johnson, and Rodger (2011) categorized the barriers against achieving educational successes by student parents (such as student mothers) into three categories: dispositional, situational, and institutional (Fairchild, 2003; Flynn et al., 2011; Hayes Nelson, 2009; Mercer, 1993). The depositional scope includes previous experiences, social perceptions, and personal, academic and occupational motives. The situational scope refers to the individual’s living situation, including poverty, rage, life conditions, and family support. The institutional scope is related to participatory barriers which are related to programmes held by training institutes (Flynn et al., 2011).However, Smith (2008) believed that, despite the wide spectrum of classifications performed in terms of facilities and barriers, these classifications are multidimensional, complex, and dependent on one another (Smith, 2008). Moreover, these factors are of wide ranges rather than being exclusive, depending on the researchers’ research context (Mullen, 2010; Ribeiro, Gonçalves, Quintas, Monteiro, & Fragoso, 2013). In this regard, McGhee, Burns, and Wood (2012) stipulate that parent students are well aware of their environment and recognize barriers to their success. Undertaking research is one method of identifying and breaking these barriers and making the necessary interventions based on evidence-based and research-specific decision making (Ellen et al., 2014; McGhee et al., 2012).

According to the findings of this study, the different aspects of self-efficacy facilitate the simultaneous management of motherhood and studentship roles. Self-efficacy means an understanding of each individual’s abilities to achieve specific goals, which influence the individual’s thoughts, feelings, and performances (Hassankhani, Mohajjel Aghdam, Rahmani, & Mohammadpoorfard, 2015). Ghasem and Hosein-chari (2013) believed that a strong sense of self-efficacy not only facilitated performance, but also helped individuals stand up to and resist failure (Ghasem & Hosein-chari, 2013). Price (2006) stated that married couples performed better, even though being married increased a woman’s duties and responsibilities (Price, 2006). Goldrick-Rab, Minikel-Lacocque, and Kinsley (2011) also believed that a sense of adulthood and responsibility towards others increases self-efficacy in the parents of U.S. students (Goldrick-Rab et al., 2011). Leaman (2015) quoting from Cross stated that mature students knew their interests in the student role. Therefore, they had a greater tendency to face life challenges (Leaman, 2015). These findings are consistent with the findings of the current study regarding the sense of self-efficacy among student mothers.

Getting support was another factor influencing the simultaneous management of the roles of mother and student. Obviously, finding compatibility between the identities of mother and student can be challenging. Familial support can empower student mothers and reduce the impact of the student role on their maternal and family life, especially on the children (Wainwright & Marandet, 2010). Previous studies have also indicated that family and friends are the most common sources of support for student mothers with family responsibilities (Xuereb, 2014). Goldrick-Rab et al. (2011) referred to the role of family members in enabling the parents of U.S. students to continue their education (Goldrick-Rab et al., 2011). In a study by Zaitawi (1999) on stress and social support among students in German universities, the researchers found that female students experienced more stress that was negatively related to social support. In fact, social support was a social process affected health and mental wellbeing which led to increased self-efficacy and the reduction of stress (Emadpour, Gholami Lavasani, & Shahcheraghi, 2016; Hamdan-Mansour & Dawani, 2008; Zaitawi, 1999).

According to the findings of the present study, near and distant relatives, especially mothers, spouses, friends, teachers, and classmates, provided support to student mothers. In line with these findings, Adofo (2013) pointed to the supportive role of students’ spouses in financial affairs, childcare, and routine domestic tasks, which reduced the workload imposed on student mothers (Adofo, 2013). The support given by university tutors, authorities, and classmates also facilitated the implementation of student mothers’ educational tasks. Moreover, the flexibility of educational schedules and the cooperation of educational staff are invaluable factors in a student mother’s education (Adu-Yeboah, 2015). According to the study by Berg and Mamhute (2013) conducted in South Africa, support from classmates was required. Data collected from pregnant women and mothers of young children indicated the importance of supportive social and educational environments (Berg & Mamhute, 2013).

According to the findings of this study, difficult social and financial conditions were barriers to the simultaneous management of the roles of mother and student (Billari & Philipov, 2004). A gender-based study showed that female students faced more problems in adapting to their study programmes, had more systematic cultural challenges and limitations, and received less support (Schmidt & Umans, 2014). Other studies also showed that inappropriate social and financial conditions adversely affected the student mother’s simultaneous management of motherhood and student roles (Billari & Philipov, 2004). Wainwright and Marandet (2010) stated that barriers to education and how they affected British students were associated with the socio-economic status (Wainwright & Marandet, 2010). A lack of family support due to family disputes was also mentioned as a barrier. Such a dispute may be the result of a husband’s dissatisfaction with the education of the student mother (Brooks, 2013). Adofo (2013) stated that one important factor affecting the achievement of married women in higher education was the expectations and attitudes of husbands (Adofo, 2013). The resistance of family members to a student mother’s education was due to a lack of belief in the importance of education for mothers (Archer, 2003; Brooks, 2013). Husbands’ attitudes can affect the level of support they provide. According to Suitor (1987), a reduction of martial satisfaction among full-time student mothers depended on changes in the woman’s role in the family and her husband’s response to these changes. Nevertheless, some student mothers received no help from their husbands as their husbands disagreed with their education (Adofo, 2013; Suitor, 1987). Iravani Shirazi (2014) believed that breaking patriarchal constraints regarding the education of females is difficult (Shirazi, 2014). The husband’s education level affects the belief about the education of females (Saroukhani, 2011). In such a society, however, the priority of family duties is emphasized. Such a perspective increases male domination over females and makes females’ education dependent upon the consent and satisfaction of men (Mosavi, 2013; Shah & Shah, 2012).

The social image of the mother caused a lack of familial support and family disputes in this study. In the 21st century, about 70% of student mothers in New York universities abandoned their education because of a lack of support from society and the family. Bullen, Kenway, and Hay (2000) believed that stigmatization was one part of the lack of support. In Australia, women’s education and training were so stigmatized that it hindered their future career options (Berg & Mamhute, 2013; Bullen et al., 2000). Recent studies on the process of women’s education have shown that when the roles of women in society increased, their traditional caring role was often preserved. In Gill et al.’s (2015) study, all British participants claimed that the primary responsibility for childcare was on the shoulders of female individuals. In fact, the impact of the family and its supportive role on students was irrefragably related to cultural expectations regarding sexual roles in the family, particularly the traditional roles of females as caregivers and males as providers (Gill et al., 2015). Similar to many Muslim countries, motherly duties in Iran have priority over all other duties (Khodami, 2015). Also, Iranian religious instructors believe that women’s education is unavoidable and should be supported, if those women accept their motherly responsibilities and work as much as males do in the social setting (Haji Esmaeili, 2016).

A discussion on the financial issue of education was raised by the participants in this study. The results of Kenny et al.’s (2007) study entitled Family and financial responsibilities for Australian adult students were consistent with the results of this study. In some cases, financial needs make mothers work while educating; this is known as a barrier to the mother’s academic success (Erk, 2013; Lenaghan & Sengupta, 2007). In Kenny et al. (2007) study, financial issues were of the most important factors affecting mother students’ abilities to complete their educations successfully (Kenny et al., 2007). Xuereb (2014) mentioned that financial stress was one of the most common reasons that British students reconsidered continuing their education (Xuereb, 2014). Moreover, parents’ employment while educating added to the challenges associated with their roles as a student, an employee, and a family member (Leaman, 2015).

The inflexible education system was another factor that hindered the simultaneous management of mother and student roles. The participants believed that the education system did not support student mothers and was not flexible towards their needs. According to Adofo (2013), academic environments in Ghana often described student mothers as problematic students. Problems caused by environmental factors were ignored and students were blamed as they had unreasonable expectations from the university (Adofo, 2013). In Trepal, Stinchfield, and Haiyasoso's (2013) review of parent students’ experiences, unpleasant experiences and the dominant negative culture that existed in educational groups sent negative messages to U.S. parent students (Trepal, Stinchfield, & Haiyasoso, 2013). The results of Trepal et al.(2013) study were consistent with the findings of this study. In line with the results of the present study, Marandet and Wainwright’s (2010) study entitled Invisible experiences found that one sixth of British interviewees pointed to a lack of understanding and support by academics as their major problem (Marandet & Wainwright, 2010). According to Moreau & Kerner’s (2013) research, universities provided little support to British parent students and treated them like students without parental responsibilities (Moreau & Kerner, 2013).

It was found that the major barrier to the simultaneous management of the roles of mother and student was accompanied by physical and mental strain. The combination of maternal and academic responsibilities is challenging, brings physical and psychological pressures, and affects academic activities (Esia-Donkoh, 2014). In addition, some mother students were further employed, which could double the overall load of stress resulting from their multiple roles (Kenny et al., 2007). This study suggested that student mothers suffered from fatigue and physical stress. According to Mark’s scarcity theory, role competitions reduce individuals’ abilities to pay enough attention to both roles at the same time (Ugwu, Orjiakor, Enweruzor, Onyedibe, & Ugwu, 2016). Marandet and Wainwright (2010) stated that many British women students were concerned that the creation of a balance between education and family responsibilities led to less rest and fatigue. They believed that an attempt to fit in with college life came at the expense of students’ health (Marandet & Wainwright, 2010). The results of Goldrick-Rab et al. (2011) study showed that a lack of rest and relaxation impacted students’ health and indirectly affected their academic performance (Goldrick-Rab et al., 2011). In addition to physical strains, other studies on mental pressures affecting student mothers were in line with the findings of this study. According to Lynch (2008), student mothers were involved in a complex identity conflict and constantly managed their behaviours to comply with the images of a good mother and good student; this situation could lead to severe stress (Brooks, 2013; Lynch, 2008). Zhang (2011) believed that being a good mother created pressure for mothers. Therefore, U.S. female students’ inability to be good mothers and good students caused feelings of anxiety and hopelessness (Zhang, 2011). Brooks (2014) believed that emotional responses such as anxiety depended on the factors that made individuals feel that they made unusual choices (Brooks, 2014). The psychological pressures towards being a good mother exist not only for those mothers with dependent little children, but also for mothers of teenaged and young children. For example, some researchers believe that many of the high-risk behaviours in adolescence and pre-teen children and their mental and emotional health are dependent upon their living conditions such as time spent with the mother (Dunifon & Gill, 2013; Mendolia, 2014). Moreover, in this stage, the generation gap raises some communicational problems, making it difficult for mature parents to play the mothering role (Bojczyk, Lehan, McWey, Melson, & Kaufman, 2011). Ozmete and Bayolu (2009) believed that the relationships between parents and their teenaged or young children are full of conflict, unpleasantness, and parental stress as compared to the same relationship in the child’s younger years (Ozmete & Bayolu, 2009). On the other hand, student mothers experienced stress and mental strains regarding academic goals and aspirations. In their study that explored the experiences of female PhD students in England, Brown and Watson (2010) determined that being a mother had a great influence on education at the Ph.D. level. Also, the creation of a balance between domestic and academic life was a source of stress (Brown & Watson, 2010). The feeling of stress in academic situations causes psychological problems in students and may negatively impact their well-being and personal learning (Hjeltnes, Binder, Moltu, & Dundas, 2015).

### Limitations

The findings of this study increase the knowledge of student mothers’ experiences of motherhood. However, these findings cannot be generalized to all student mothers in other contexts and settings because of the small sample size. Future studies should be conducted in other cultures and contexts to improve the transferability of the current findings.

## Conclusions

The management of maternal and family affairs by female students in universities in which the motherhood role is not supported is a challenge. There is a need to emphasize the significance of the roles of mother and student and to provide support and education for gaining skills to play these roles. In addition, policy makers should devise strategies for bringing change to the traditional perspective that motherhood and educational responsibilities cannot be met simultaneously by one person. The structure of universities should be family friendly. Also, a discourse should be initiated to change the traditional contrast between caregiving and educational responsibilities.
